# Androgen Receptor Status Is a Prognostic Marker in Non-Basal Triple Negative Breast Cancers and Determines Novel Therapeutic Options

**DOI:** 10.1371/journal.pone.0088525

**Published:** 2014-02-05

**Authors:** Pierluigi Gasparini, Matteo Fassan, Luciano Cascione, Gulnur Guler, Serdar Balci, Cigdem Irkkan, Carolyn Paisie, Francesca Lovat, Carl Morrison, Jianying Zhang, Aldo Scarpa, Carlo M. Croce, Charles L. Shapiro, Kay Huebner

**Affiliations:** 1 Department of Molecular Virology, Immunology and Medical Genetics, Ohio State University Wexner Medical Center and Comprehensive Cancer Center, Columbus, Ohio, United States of America; 2 ARC-NET Research Centre, University and Hospital Trust of Verona, Verona Italy; 3 Department of Pathology, Hacettepe University, Ankara Turkey; 4 Department of Pathology, Roswell Park Cancer Institute, Buffalo, New York, United States of America; 5 Bioinformatics Shared Resource, Comprehensive Cancer Center, Ohio State University, Columbus, Ohio, United States of America; 6 Division of Medical Oncology and the Breast Program, James Cancer Hospital and Ohio State University Comprehensive Cancer Center, Columbus, Ohio, United States of America; University of Torino, Italy

## Abstract

Triple negative breast cancers are a heterogeneous group of tumors characterized by poor patient survival and lack of targeted therapeutics. Androgen receptor has been associated with triple negative breast cancer pathogenesis, but its role in the different subtypes has not been clearly defined. We examined androgen receptor protein expression by immunohistochemical analysis in 678 breast cancers, including 396 triple negative cancers. Fifty matched lymph node metastases were also examined. Association of expression status with clinical (race, survival) and pathological (basal, non-basal subtype, stage, grade) features was also evaluated. In 160 triple negative breast cancers, mRNA microarray expression profiling was performed, and differences according to androgen receptor status were analyzed. In triple negative cancers the percentage of androgen receptor positive cases was lower (24.8% *vs* 81.6% of non-triple negative cases), especially in African American women (16.7% *vs* 25.5% of cancers of white women). No significant difference in androgen receptor expression was observed in primary tumors *vs* matched metastatic lesions. Positive androgen receptor immunoreactivity was inversely correlated with tumor grade (p<0.01) and associated with better overall patient survival (p = 0.032) in the non-basal triple negative cancer group. In the microarray study, expression of three genes (*HER4*, *TNFSF10*, *CDK6*) showed significant deregulation in association with androgen receptor status; *eg* CDK6, a novel therapeutic target in triple negative cancers, showed significantly higher expression level in androgen receptor negative cases (p<0.01). These findings confirm the prognostic impact of androgen receptor expression in non-basal triple negative breast cancers, and suggest targeting of new androgen receptor-related molecular pathways in patients with these cancers.

## Introduction

The prognostic role of hormone receptors has widespread acceptance in the management of breast cancer. In spite of this, androgen receptor (AR) dysregulation and its therapeutic value has only recently been investigated in this group of neoplasms [1], [2], [3]. Over 70% of human breast cancers express AR [4], [5], [6], [7], and AR positive cases are significantly associated with a low risk of tumor recurrence and patient death [5], [8], [9], [10], [11]. Recent *in vitro* studies pinpointed the significant influence of estrogen receptor α (ER) status on androgen-dependant cell growth stimulation [5], [12], [13], [14], [15]: androgens tend to inhibit the growth of AR-positive and ER-positive breast cancer cells but stimulate the growth of AR-positive and ER-negative cells. *In vivo* studies further corroborated this finding. In ER-positive luminal breast cancers, AR has a growth inhibitor role but AR signaling may promote growth of a subset of ER-negative AR-positive breast cancers [1], [2], [9], [10], [16]. On these bases, clinical trials (ClinicalTrials.gov) have been established focusing on AR targeting in ER-negative cases, such as triple negative breast cancers (TNBCs) [13], [17].

TNBCs are clinically defined by the lack of expression of ER, progesterone receptor (PR), and the absence of amplification or overexpression of HER2 [18], [19], [20]. This group of tumors accounts for 15% to 20% of newly diagnosed breast cancer cases [18]. In general, patients with TNBC present with larger tumors of higher grade, increased number of involved nodes, and poorer survival compared with other cancer subtypes. Mounting evidence indicates that TNBC is a highly heterogeneous disease on a molecular level [19]. Treatment of TNBC patients has been challenging due to this heterogeneity and the absence of well-defined molecular targets.

AR has been detected in only 25%–35% of TNBCs [13], [17], [21], [22], [23] and AR negativity has been associated with a shorter disease-free interval and overall survival as compared to AR-positive TNBCs [13], [17], [21], [22], [24], [25], [26], [27]. Moreover, decreased AR expression has been associated with the occurrence of distant metastasis [17], [28].

Stratification of the heterogeneous group of TNBCs into subclasses using new markers will identify new screening methods, prognostic factors, and perhaps targets for personalized therapies. A five-marker immunohistochemical panel (comprising ER, PR, HER2, EGFR, and cytokeratin 5/6 [CK5/6]) has been introduced to subclassify TNBCs into two major prognostic classes: Core Basal (EGFR and/or CK5/6 positive) and 5 negative (5NP) tumors [29]. Little preliminary data is available concerning AR status in the different TNBC subtypes [24], [30]. In this study, we investigated AR expression by immunohistochemical staining in 678 breast cancers, including 396 TNBCs. Data were further evaluated according to clinical (race, survival) and pathological (TNBC subtyping, staging, grading) features. In 160 TNBCs of the series, mRNA microarray expression profiling was performed, and differences associated with AR expression status were analyzed. We further supported the notion that AR is a prognostic marker in TNBC tumors and demonstrated for the first time that the AR has a prognostic impact only in non-basal or 5NP tumors. AR-negative cases were characterized by a specific mRNA profile and novel targetable markers were identified.

## Materials and Methods

### Ethic statement

All the fixed and anonymized samples were received anonymously and processed for RNA and immunohistochemical analyses at the Department of Molecular Virology, Immunology and Medical Genetics of the Ohio State University (OSU). IRB-approved protocol for this research (OSU ethics committee: #2009C0004) linked clinical features, treatment and outcome data of breast cancer patients in the OSU National Comprehensive Cancer Network breast cancer database/tumor registry and the Roswell Park Cancer Institute (RPCI) with archival breast cancer pathology specimens stored in the OSU and RPCI Tissue Archive Service and provided de-identified clinico-pathological information. The institutional ethics committees waived the need for informed consent.

### cDNA microarray analysis

The Oncomine database and gene microarray analysis tool, a repository for published cDNA microarray data (www.oncomine.org) [31] was explored (15th July 2013) for *AR* mRNA expression in The Cancer Genome Atlas (TCGA) breast cancer series. Oncomine algorithms were used for the statistical analysis of the differences in *AR* mRNA expression.

### Patients

**Figure 1:**
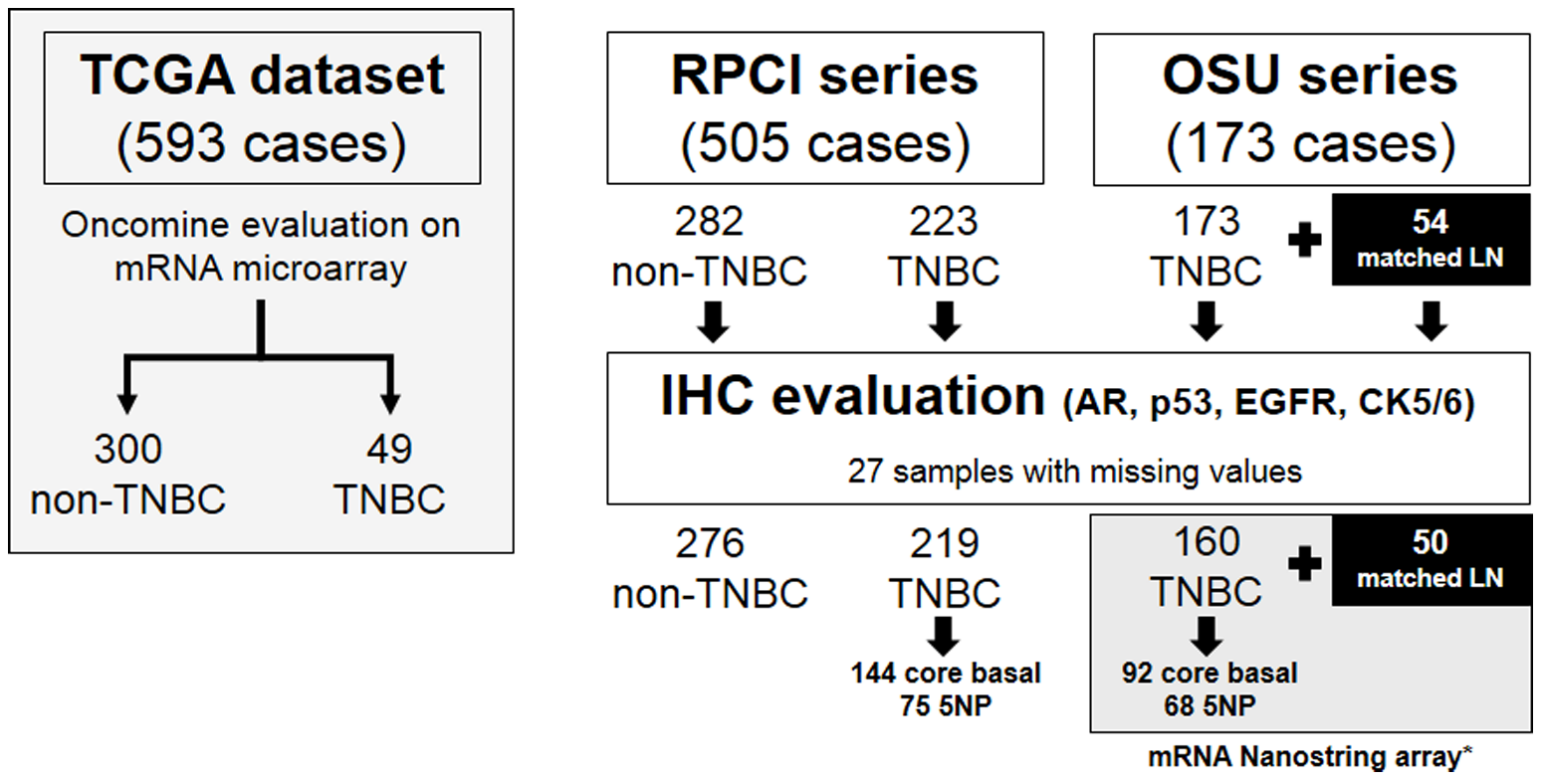
Overview of the cohorts examined and molecular tests applied. * All the 54 lymph node metastases were analyzed by Nanostring mRNA array.

Institutional female breast cancer cohorts from: i) the OSU National Comprehensive Cancer Network breast cancer database/tumor registry, and ii) RPCI, were used in this study. For all cohorts, tumor paraffin blocks were assigned an anonymous unique identifier linked to databases that contained pathological, and clinical data. Outcome data were available for the OSU series. Tissue microarrays (TMAs) were constructed and each TMA contained 1-mm cores sampled from representative paraffin blocks from each patient. To avoid TMA-related underestimation of tumor heterogeneity, triplicate TMA blocks (and therefore three samples per patient) were considered. The OSU TNBC series was also used for RNA preparation. ER, PR, and HER2 (both immunohistochemical and FISH) status were retrospectively obtained from the original pathological reports. Institutional review board approval was obtained for the use of patient blocks at each institution. Previously assembled TMAs comprehensive of 173 TNBCs constituted the OSU cohort [19]. For 50 of the primary tumors there were also fixed matched lymph node metastatic lesions. The RPCI TMAs comprised a series of 505 primary breast cancer specimens. Tissues of patients who received adjuvant and/or neoadjuvant chemotherapy were included in the analysis. TNBC patients received neoadjuvant and/or adjuvant therapy in 11.4% and 85.4% of cases, respectively; 89.0% of treatment regimens were based on the use of anthracycline and/or taxanes. The clinical and pathologic characteristics of the series (OSU plus RPCI) are summarized in **Table 1** and in **Figure 1**.

**Table 1:** Clinico-pathological features of the considered series.

	Total (n = 678)	TNBC (n = 396)	Core Basal (n = 236)	5NP (n = 143)	*p*-value (Core Basal *vs* 5NP)	Non-TNBC (n = 276)	*p*-value (TNBC *vs* non-TNBC)
**Age** (mean±SD)	54.9±13.2	53.8±13.4	54.9±13.9	53.4±12.7	n.s.	56.4±12.8	n.s.
**Race**					n.s.		n.s.
Whyte	511 (75.4%)	332 (83.8%)	194 (82.2%)	125 (87.4%)		175 (63.4%)	
African American	153 (22.6%)	55 (13.9%)	36 (15.3%)	15 (10.5%)		96 (34.8%)	
Others	12 (1.8%)	7 (1.8%)	4 (1.7%)	3 (2.1%)		5 (1.8%)	
Missing	2 (0.3%)	2 (0.5%)	2 (0.8%)	0 (0.0%)		0 (0.0%)	
**Histotype**					n.s.		n.s.
ductal	506 (74.6%)	311 (78.5%)	194 (82.2%)	104 (72.7%)		191 (69.2%)	
lobular	44 (6.5%)	10 (2.5%)	2 (0.8%)	7 (4.9%)		33 (12.0%)	
others	121 (17.8%)	68 (17.2%)	39 (16.5%)	26 (18.2%)		52 (18.8%)	
missing	7 (1.0%)	7 (1.7%)	1 (0.4%)	6 (4.2%)		0 (0.0%)	
**Stage**					n.s.		n.s.
I	164 (24.2%)	114 (28.8%)	60 (25.4%)	48 (33.6%)		50 (18.1%)	
II	323 (47.6%)	196 (49.5%)	119 (50.4%)	68 (47.6%)		124 (44.9%)	
III	110 (16.2%)	66 (16.7%)	43 (18.2%)	21 (14.7%)		42 (15.2%)	
IV	6 (0.9%)	5 (1.3%)	3 (1.3%)	2 (1.4%)		1 (0.4%)	
Missing	75 (11.1%)	15 (3.8%)	10 (4.2%)	4 (2.8%)		59 (21.4%)	
**Grading**					n.s.		<0.001
G1	37 (5.5%)	7 (1.8%)	2 (0.8%)	5 (3.5%)		30 (10.9%)	
G2	137 (20.2%)	38 (9.6%)	23 (9.7%)	12 (8.4%)		97 (35.1%)	
G3	481 (70.9%)	345 (87.1%)	206 (87.3%)	125 (87.4%)		133 (48.2%)	
missing	23 (3.4%)	6 (1.5%)	5 (2.1%)	1 (0.7%)		16 (5.6%)	

### Definition of Breast Cancer Subtypes

Only nuclear reactivity was taken into account for ER, and PR, irrespective of the staining intensity, whereas only an intense and complete membrane staining in >10% of the tumor cells qualified for HER2 overexpression (3+) [32], [33]. FISH assay for HER2 was performed in selected cases (i.e., those with 2+ immunoreactivity) as previously described [34]. Triple-negative tumors were defined as tumors that were both ER and PR negative and in which HER2 was not amplified or overexpressed. HER2-positive (HER2+) tumors included both ER-positive and ER-negative tumors and showed HER2 amplification or overexpression. ER/PR-positive/HER2-negative (ER/PR+) tumors were defined as ER-positive and/or PR-positive, and HER2-negative [35].

### Immunohistochemistry

Immunohistochemical reactions were performed automatically (Dako Autostainer immunostaining system; Dako) for CK5/6 (D5/16 B4; Dako; 1∶100), EGFR (2-18C9; Dako; 1∶100), AR (F.39.4.1; BioGenex; 1∶100), and p53 (mouse (Dako, M7001; 1∶100). P53 was considered in the analysis as a quality control marker of the immunohistochemical reactions because it is a driver gene in TNBCs [20]. Appropriate positive and negative control tissues were run concurrently.

The expression of CK5/6 was cytoplasmic, the expression of EGFR was both cytoplasmic and membranous, expression of AR and p53 was nuclear. Cytoplasmic expression in ≥10% of tumor cells for CK5/6, membranous staining in ≥10% of tumor cells for EGFR, and nuclear staining in ≥5% of tumor cells for AR and ≥50% for p53 were accepted as positive, as previously described [36], [37].

TNBCs were divided into subtypes of breast cancer as defined by their IHC profiles as basal-like triple negative (Core Basal; negative for ER, PR, and HER-2 and positive for CK5/6 and/or EGFR), and five negative (5NP; negative for ER, PR, HER-2, CK5/6, and EGFR). Slides were scored independently by three pathologists (SB, CI, MF) blinded to breast cancer subtype; one pathologist (MF) converted scores to numbers, selected cutoff values for each marker and entered data into Excel files.

### nanoString nCounter mRNA profile analysis

RNA was isolated from formalin-fixed paraffin-embedded tissue of 160 TNBCs, 59 tumor-associated, adjacent normal and 54 matched lymph node metastatic tissues, using the Recover ALL kit (Ambion). RNAs were profiled for mRNA expression using the nanoString nCounter system (nanoString, Seattle, Washington, USA) in the Nucleic Acid Shared Resource of The Ohio State University Comprehensive Cancer Center. The nanoString GX Human mRNA Cancer Reference panel, that includes tags specific for 230 cancer-related mRNAs (http://www.nanostring.com/products/gene_expression_panels.php), was used. The mRNA microarray expression data have been submitted to the Gene Expression Omnibus (GEO) with accession number GSE 41970.

### Statistical Analysis

Not all marker or clinical data were available on all subjects, and percentages refer to cases for which data for a specific variable were available. Associations between categorical variables were evaluated using chi-square or Fisher exact tests.

Kaplan–Meier and a multivariate Cox proportional hazards model were used to evaluate overall survival (OS), where differences in distributions were evaluated based on clinical characteristics and marker expression. Only patients who met the following inclusion criteria were considered: i) <75 years of age, to exclude potential bias of fatal co-morbidities more prevalent in elderly patients; ii) ductal and/or lobular histotype, to avoid the influence of dedifferentiated subtypes on prognosis. A final series of 153 patients were included in the analysis. The P-values reported in relation to patient survival correspond to log rank tests unless otherwise noted.

To investigate the differences among the gene expression profiles detected by the nanoString GX Human mRNA Cancer Reference Kit, we performed hierarchical clustering using dysregulated genes according to IHC AR status. Two-dimensional average-linkage hierarchical clustering of a Spearman rank correlation similarity matrix of the two groups (AR positive *vs* AR negative) was performed. All gene expression analyses were performed using R software (version 2.13.0).

For mRNA studies, all fold-changes associated with these analyses are represented in log2 scale.

## Results

### Androgen receptor expression is down-regulated in TNBCs

**Figure 2:**
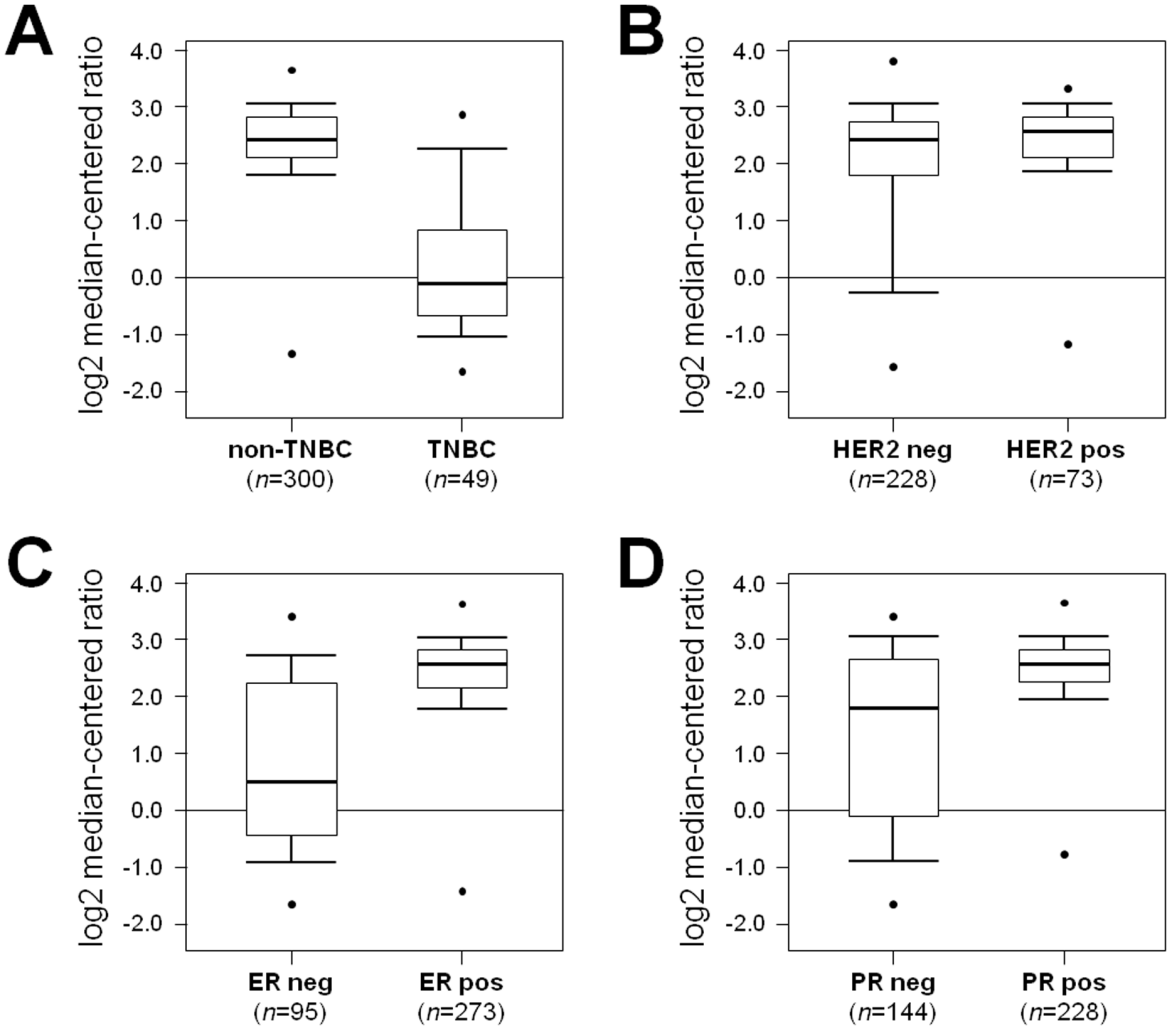
Androgen receptor mRNA expression is down-regulated in TNBC cases. Expression microarray results of the TCGA consortium data set was analyzed, and statistical significance was calculated using the Oncomine website (www.oncomine.org). Box plots show differences in mRNA expression between the different classes according to TNBC (**A**), HER2 (**B**), ER (**C**), and PR (**D**) status. Data are presented as box plot distribution (Line within the boxes =  median value).

The Oncomine database and gene microarray data analysis tool enabled the meta-analysis of gene expression in the breast cancer TCGA microarray studies [38] (**Figure 1**). In the analysis, we considered the expression levels of AR in the major different subtypes of breast cancer. AR was significantly down-regulated in TNBC samples (**Figure 2A**; *p*<0.001), and up-regulated in HER2 positive cases (**Figure 2B**; *p* = 0.025), as well as in ER and PR positive cases (**Figure 2C–D**; *p*<0.001 both).

The Oncomine mRNA data were confirmed by immunohistochemical analysis of AR expression on breast cancer TMAs obtained from two independent Institutional cohorts (OSU and RPCI). A total of 678 breast cancer specimens were evaluated. The series comprised 506 ductal carcinomas and 44 lobular carcinomas (128 cases with different histotype or missing data). The ethnic distribution was of 474 white and 145 African American women (59 patients were of another race or missing data). The immunohistochemical profile categorized samples in 396 TNBCs and 276 non-TNBC cases (in 6 cases the data on HER2 expression was missing).

**Figure 3:**
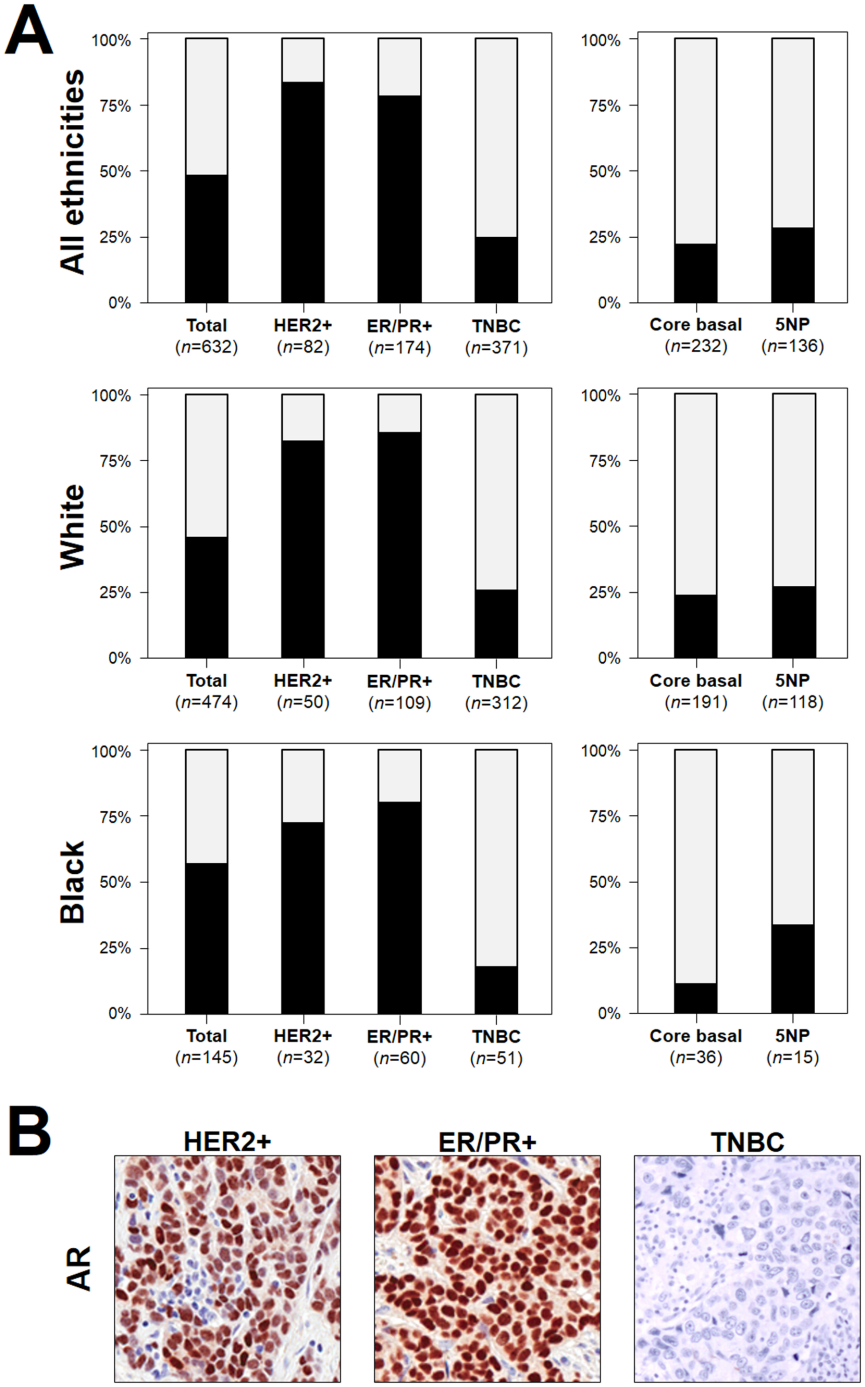
Androgen receptor expression is low in TNBC cases. AR expression distribution in the considered cancers. (**A**) Nuclear AR staining was significantly lower in TNBC specimens than in HER2-positive and ER/PR-positive tissues (p < 0.001). The trend toward expression differences among different ethnicities was not significant. Among TNBCs, 5NP cancers showed a higher frequency of AR expression (*p* = ns). Numbers represent TMA cores available for the analysis. Black  =  AR positive cases, gray  =  AR negative cases. (**B**) Representative images of AR immunostaining in the three cancer subtypes (HER2-positive, ER/PR-positive, and TNBC). (Original magnification, 200×)

**Figure 4:**
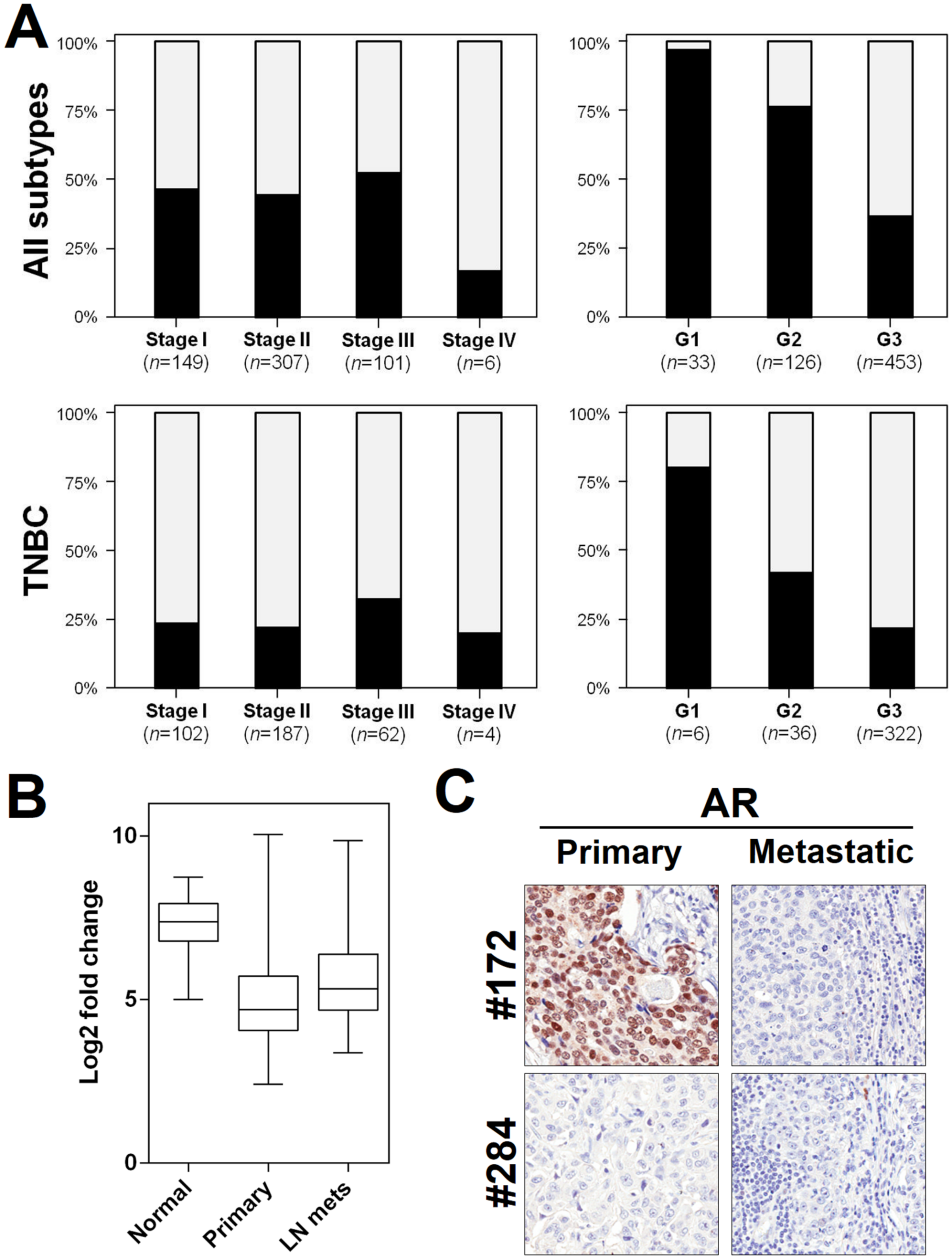
Androgen receptor status is associated with tumor grade and is consistent among primary and secondary lesions. AR expression distribution according to tumor stage and grade (**A**). AR was lower in high grade tumors in both the whole series and in the TNBC subgroup (*p*<0.01 both), but was consistently distributed among the different tumor stages. Black  =  AR positive cases, gray  =  AR negative cases. (**B**) AR mRNA expression is down-regulated in TNBC primary tumors and lymph node metastases. (**C**) Representative images of AR immunostaining in two matched primary tumors and lymph node metastases. Case #172 showed a positive primary neoplasm and a negative metastatic lesion. Case #284 is AR negative in both neoplastic lesions. (Original magnification, 200×)

HER2-positive and ER/PR-positive carcinomas showed higher prevalence of AR positive cases than TNBCs (**Figure 3**, **Table 2**). Prevalence of AR-positive cases was lower in high grade tumors (**Figure 4A**, *p*<0.01), but was consistently distributed among the different tumor stages (**Figure 4A**); 98.4% (300/305) of AR positive cases (98.6% in non-TNBC and 97.8% in TNBC) showed ≥10% AR expression.

**Table 2:** Immunohistochemical profiles of the considered series.

Protein	Total (n = 678)	TNBC (n = 396)	Core Basal (n = 236)	5NP (n = 143)	*p*-value (Core Basal *vs* 5NP)	Non-TNBC (n = 276)	*p*-value (TNBC *vs* non-TNBC)
AR	305/632 (48.3%)	92/371 (24.8%)	50/229 (21.8%)	38/136 (27.9%)	n.s.	209/256 (81.6%)	<0.001
p53	268/639 (41.9%)	202/377 (53.6%)	129/232 (55.6%)	70/138 (50.7%)	n.s.	66/256 (25.7%)	<0.001
EGFR	249/648 (38.4%)	210/381 (55.1%)	207/235 (88.1%)	0/143 (0.0%)	<0.001	37/262 (14.1%)	<0.001
CK5/6	98/646 (15.2%)	89/385 (23.1%)	88/236 (37.3%)	0/143 (0.0%)	<0.001	9/256 (3.5%)	<0.001

Considering TNBC cases only, AR-positive cases were less frequent in the African American cancer cohort in comparison to cancers of white patients (16.7% vs 25.5%); the 5NP group showed AR-positive cases more frequently than Core Basal tumors (*p* = ns).

As previously described [18], [19], [20], TNBCs were more frequently p53, EGFR and CK5/6 positive in comparison to other breast cancer subtypes (**Table 2**). No differences were observed in p53 expression between Core Basal and 5NP cases, whereas 5NP tumors were EGFR and CK5/6 negative by definition.

### Androgen receptor in matched primary and metastatic TNBC lesions

To further test the feasibility of anti-AR therapy in TNBC metastatic disease, we studied AR status in TNBC lymph node metastases by mRNA and protein expression analyses. A series of 160 tumor samples, 59 tumor-associated adjacent normal, and 54 matched lymph node metastatic tissues were evaluated for RNA expression using the nanostring platform. AR mRNA was significantly down-regulated in primary and metastatic TNBC samples in comparison to normal breast tissue (both p<0.001; **Figure 4B**), and significant up-regulation was observed in metastatic samples in comparison to primary cancers (*p* = 0.02). Matched cases (primary *vs* metastatic sample) reveal comparable levels of mRNA expression (p = ns).

For 50 cases of the OSU TNBC series, a matched lymph node metastatic lesion was available for immunophenotyping. AR status in lymph node metastases was similar to that observed in the primary cancers (36/50; *p* = 0.03). In three cases the metastasis became AR positive, whereas in 11 the metastatic lesion was negative with a concurrent AR positive primary tumor (**Figure 4C**).

### Androgen receptor is a prognostic marker for non-basal TNBCs

**Figure 5:**
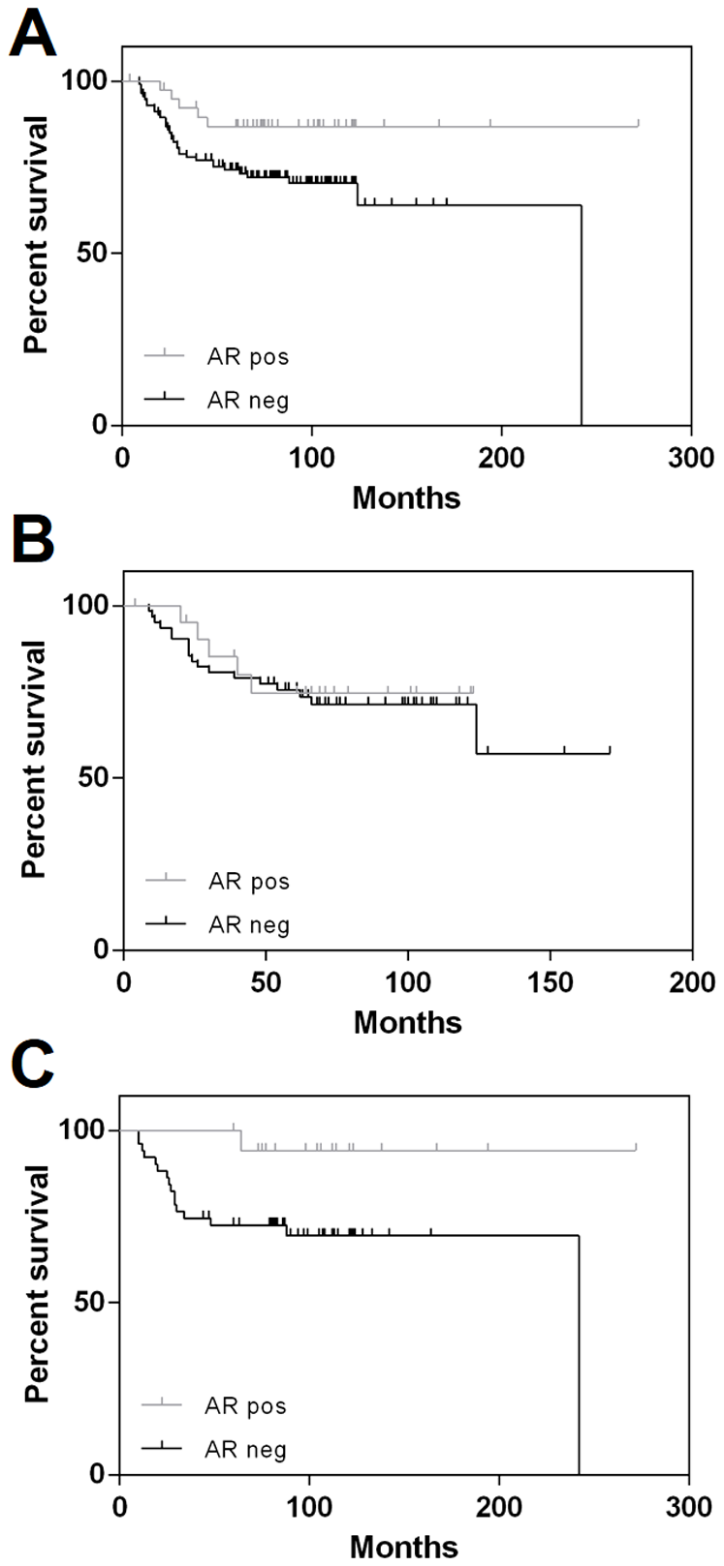
Androgen receptor status correlates with better prognosis in 5NP TNBCs. Correlation between AR expression status and overall survival in TNBCs (*n* = 153; *p* = 0.032) (**A**), Core Basal TNBCs (*n* = 84; *p* = ns) (**B**), and 5NP TNBCs (*n* = 69; *p* = 0.026) (**C**) patients.

A series of 173 TNBC patients was considered to assess the prognostic value of AR immunohistochemical evaluation in TNBCs for overall patient survival. AR immunohistochemical positivity was associated with better overall survival (*p* = 0.032) in TNBCs (*n* = 153; **Figure 5**); no significant difference in survival was observed in the Core Basal group (*p* = ns; *n* = 86), whereas AR expression identified a subclass of patients with better overall survival among 5NP tumors (*n* = 69; *p* = 0.026). No association of AR expression with and good overall patient prognosis was observed in multivariate Cox hazard analysis (the analysis considered TNM, grading, histotype, and TNBC subtypes).

### AR status identifies new candidate therapeutic approaches for TNBC

Emerging preclinical and clinical data suggest that AR may serve as a therapeutic target in certain difficult-to-treat breast cancer subtypes, such as TNBC [23]. Thus, identification of novel targetable biomarkers in AR-negative (and therapeutically orphan) TNBC cases is of primary importance.

**Figure 6:**
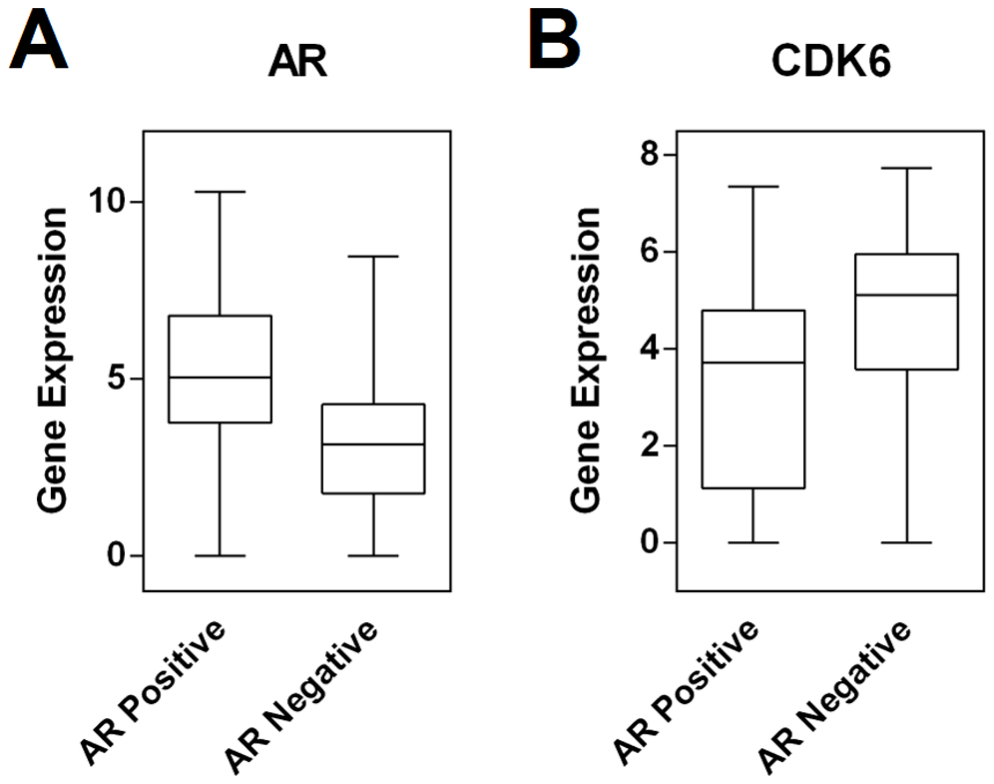
Androgen receptor expression status defines novel potential therapeutic targets. mRNA expression profiling of 160 TNBC cases was dichotomized according to their AR immunohistochemical status (AR negative *vs* AR positive). As expected, AR mRNA was significantly up-regulated in AR-positive cases (logFC 2.33; p<0.01; **A**). Among the deregulated mRNAs, CDK6 showed significantly higher expression in AR-negative cases (logFC -1.16; p<0.01; **B**).

mRNA expression profiles of 160 TNBC cases were stratified according to AR immunohistochemical status (AR negative *vs* AR positive; 6 cases had missing AR data and were ruled out). As expected, AR mRNA was significantly up-regulated in AR-positive cases (logFC 2.33; p<0.01). Three mRNAs were significantly different in the two groups, two up-regulated genes in AR-positive cases were HER4 (logFC 0.82; p<0.01), and TNFSF10 (logFC 1.06; p<0.01). CDK6 showed a significantly lower expression in AR-positive cases (logFC -1.16; p<0.01) (**Table 3**; **Figure 6**).

**Table 3:** Differentially expressed genes in TNBCs according to AR status.

Gene ID	*p*-value	Adjusted *p*-value	AR-positive	AR-negative	log2FC
*AR*	<0.01	<0.01	5.24	2.90	2.33
*ERBB4*	0.001	0.02	0.99	0.18	0.82
*TNFSF10*	0.001	0.02	8.51	7.45	1.06
*CDK6*	0.002	0.05	3.24	4.39	−1.16

## Discussion

TNBC is a heterogeneous disease that is highly variable with respect to its biology, etiology, and treatment options [18]. Because TNBCs are more likely to be poorly differentiated, these cancers have a more aggressive clinical course [18]. Moreover, due to the lack of known specific therapeutic targets, standard treatment regimens have not been established, and, as a result, TNBC mortality remains high [18], [23]. Thus, new prognostic indicators and approaches for treatment of TNBC are needed.

In recent years, numerous pathways of interest in TNBC carcinogenesis have been studied, including AR signaling [13], [17], [21], [22], [24], [25], [26], [27]. Several studies have reported a significant association between AR status and TNBC patient outcome, but the clinico-pathological significance of AR expression among TNBC subtypes warrants further investigation [3], [13], [17], [21], [22], [24], [25], [26], [27], [39], [40].

Gene expression profiling has recently subclassified TNBC into different prognostic classes [19], [29], [41]. In this context, our group demonstrated that specific miRNA expression signatures characterize and contribute to the phenotypic diversity of TNBCs [19]. The five-marker immunohistochemical panel comprising ER, PR, HER2, EGFR, and CK5/6 is the most widely applied subcategorization, and stratifies TNBCs into Core Basal and 5NP tumors [29]. No significant difference was observed in the two subclasses according to AR expression, though a trend toward over-expression was observed in 5NP cases.

In this study, we applied a cut-off value of >10% for EGFR and CK5/6 assessment based on our previous experience [36], and on mRNA/miRNA data which confirm the molecular clusterization of these two groups according to such an immunohistochemical evaluation (Gasparini P, et al. microRNA expression profiling identifies a 4-microRNA signature as a novel diagnostic and prognostic biomarker in triple negative breast cancer. Manuscript submitted). Other investigators have suggested a cut-off value of >5%. In their seminal work, Sutton and colleagues [42] found that 94.5% of TNBCs are core basal if considering the 5% limit. In our series, 12 and 6 cases could be reclassified as EGFR and CK5/6 positive, respectively. In this circumstance, 10 5NP cases become core basal-type, which means an overall core basal prevalence of 65% (246 core basal of 379 TNBC) instead of 62% (236 core basal of 379 TNBC), which did not significantly affect AR prevalence among the groups. Differences among our and Sutton's results could be related to the different applied antisera. However, additional combined immunohistochemical-microarray studies should explore this point.

No significant difference in AR expression was observed in primary tumors in comparison to the matched metastatic samples. However, the finding that eleven AR-negative metastatic samples coexisted with AR-positive primary tumors suggests that AR loss could be associated with the metastatic process. This was further supported by the fact that p53 expression was consistent among the matched primary/metastasis pairs (i.e. both primary and metastasis negative or both positive) in all 11 cases (data not shown). A discrepancy in AR status between a primary and metastatic lesion could significantly affect AR-targeted therapeutic approaches, and should be further evaluated in larger series of matched primary and metastatic TNBC lesions.

From the prognostic point of view, AR immunoreactivity was associated with better overall patient survival (p = 0.032). Unfortunately, this result could not be confirmed by multivariate analysis. This could be related to the relatively small series of analyzed samples (i.e. *n* = 153) and to the low prevalence of AR positive cases in this specific breast cancer subtype. In keeping with our findings, other groups demonstrated a trend for improved overall survival in AR positive cases [24]. Whether this difference is indicative of a more indolent nature or whether it reflects sensitivity to TNBC-specific treatments is still unclear; none of the patients whose tumors were analyzed received antiestrogen or antiandrogen therapy.

Of interest, a significant difference was observed among Core Basal and 5NP tumors. Core Basal TNBCs have been reported to have a worse prognosis and this is true also in our series (submitted data). In contrast to recent data from Thike and colleagues [24], AR status did not modify Core Basal patient prognosis, suggesting that a multi-Institutional series of cases with definition of TNBC subtypes should further investigate implications of AR deregulation n in this specific cancer subset.

AR-targeting has been introduced recently as a novel therapeutic option in TNBC [22], and a phase II trial of Bicatulamide (Casodex, AstraZeneca; nonsteroidal anti-androgen) treatment is ongoing in women with advanced AR+/ER−/PR− breast cancer. The preliminary results of this trial suggest a benefit [13].

However, the low prevalence of AR positive cases prompts the search for alternative targetable pathways. Thus, we performed mRNA microarray expression profiling in 160 TNBC tumors, and looked for differentially expressed genes according to AR status. Three genes (i.e. *HER4*, *TNFSF10*, *CDK6*) showed significant deregulation of expression. CDK6 showed significantly higher expression in AR negative cases (p<0.01). Highly specific CDK4/6 inhibitors have been developed recently and represent a viable mechanism for systemic activation of the RB pathway [43], [44], [45]. The inhibition of CDK4/6 blocks DNA synthesis by prohibiting cell cycle progression from G1- to S-phase, resulting in a potent cytostatic effect that is dependent on a functional RB pathway [44], [46]. Tumors that are RB-deficient express exceedingly high levels of p16ink4a. The expression levels of p16ink4a and RB status were suggested to be useful prospectively to evaluate the response to CDK4/6 inhibitors [45]. Our study suggests that in TNBC, AR expression level may additionally be used to predict the response to CDK4/6 inhibitors. Targeting CDK4/6 in AR negative TNBC may be beneficial to the clinical outcome of the patients through the inhibition of cellular proliferation. Pharmacological CDK4/6 inhibition in combination with anthracycline-based chemotherapy has been tested recently in a TNBC model [47] but not in patients stratified by AR status.

In conclusion, these findings further support the prognostic impact of AR in TNBC. The varying prevalence of AR expression in the TNBC subtypes emphasized their phenotypic and molecular heterogeneity. Further efforts should investigate i) CDK6-targeting efficacy in AR-negative cases; ii) the role of AR deregulation in Core Basal and 5NP TNBC subtypes; iii) the diagnostic and therapeutic impact of AR assessment in clinical practice.
